# A Case Series: Herpes Simplex Virus as an Occupational Hazard

**DOI:** 10.1111/j.1708-8240.2011.00469.x

**Published:** 2011-08-30

**Authors:** William D Browning, James P McCarthy

**Affiliations:** * Indiana University School of DentistryIndianapolis, IN, USA; † Quadex Pharmaceuticals, LLCMidvale, UT, USA

## Abstract

**Statement of the Problem:**

Herpes labialis infections are common and present a serious risk to the dental team.

**Purpose of the Study:**

The purpose is to make dentists aware of the risks involved with treatment of patients with active herpes labialis. In addition, evidence-based risk-management strategies are presented.

**Methods and Materials:**

The incidence and natural history of herpes simplex virus type 1 (HSV-1) are reviewed. Four previously unreported case histories are presented to illustrate the impact common sequelae of HSV-1 can have on the dental team. The differences between HSV-1 and the blood-borne diseases which are the focus of universal precautions are discussed. In particular, the highly contagious, highly transmissible nature of HSV-1 and its transmission through aerosols are highlighted. Finally, the need to include protection against aerosols in the profession's understanding of universal precautions is noted.

**Results:**

The authors suggest limiting the treatment of patients with active lesions to urgent care only, and treating active HSV-1 lesions to reduce time of healing. For four common clinical situations involving HSV-1 infections, evidence-based methods for protecting the dental team and the patient from cross-contamination are also presented.

**Conclusion:**

While it is clear that the treatment of patients with active herpes labialis lesions increases risk of cross-infection, there are good protocols for controlling this risk.

**CLINICAL SIGNIFICANCE:**

By bringing common vectors of cross-infection to light and providing evidence-based protocols for preventing them, this article provides practitioners with positive steps that can be taken for controlling the risk of spreading herpes infections to the dental team.

(J Esthet Restor Dent 24:61–67, 2012)

## PURPOSE

Four previously unreported case histories are presented with the purpose of making dentists aware of the risks involved with treatment of patients with active herpes labialis. In addition, evidence-based risk-management strategies are presented.

## INTRODUCTION

While reports vary, about 85% of the US population is infected with herpes simplex virus type 1 (HSV-1).^1^–^3^ Infection tends to occur in one of two distinct timeframes: because of the nonfastidious nature of children, childhood infection is predominant. A second wave of infections tends to occur in adolescence.^2^

The primary lesion begins with a prodromal phase, progresses to papule, then to a vesicle, and finally to a full lesion. Healing takes place later via soft scab followed by hard scab. Following the primary infection, 40% of people experience recurrences. These are typically less severe.

Recurrences are mediated by the virus entering a dormant phase. During primary infection, the virus infects epithelial cells until it comes in contact with a sensory nerve. It then sheds its lipid coat, and the DNA core migrates down the nerve axon and goes latent in the nerve cell body. Following a stimulus such as ultraviolet B or febrile illness, the DNA core migrates back to the sensory neuron and begins the infection process anew.

Recent research^4^ indicates that the other 60% of people have several silent recurrences in which the infection is controlled by Cluster of differentiation 8 (CD8+) and Cluster of differentiation 4 (CD4+) T cells before clinical symptoms can be detected. Thus, it appears that the 40% who suffer recurrent lesions may have weaker cell-mediated immunity.

HSV-1 is a threat to the dental team in that it can also affect the skin (herpetic whitlow) and the cornea (ophthalmic keratitis). Here again, if these areas are the site of a primary herpetic infection, symptoms are exaggerated. More typically, these infections follow a primary oro-facial infection. In this case, the symptoms are attenuated, but there is no evidence that primary infection provides protection against acquisition of the virus at these other sites.

One of the authors (JM) is a virologist who has provided many lectures on HSV-1 to dental teams around the country. As a result, he has been contacted by many dentists over the years for counsel in handling infections and other issues involving HSV-1. The four cases described below represent an interesting cross-section of cases upon which he has consulted over the years.

## CASE HISTORIES

### Case #1—Herpes Whitlow on the Arm and Hand of a Dentist

**FIGURE 1:**
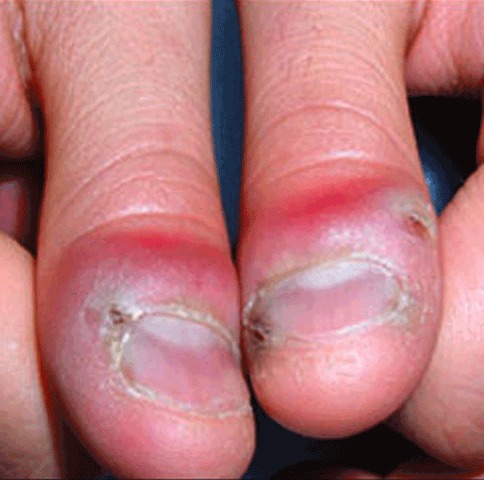
Representative image of herpetic whitlow involving the fingers. Image reprinted with permission (image is in public domain).

A dentist was about to leave for a long dental symposium. Just prior to leaving for the airport, he took a call from a patient with a need for urgent care. The dentist met the patient in his office, which was on the way to the airport. The patient presented with an open herpes lesion. Under pressure not to miss his flight, the dentist treated the patient's dental condition without first donning gloves and a long-sleeved lab coat. About 4 days later, the dentist was hospitalized with high fever and lesions accompanied by severe pain in the hand and right arm (see Figure 1).

### Case #2—Herpes Whitlow on the Arm and Hand of a Dentist

A dentist provided urgent care to a patient with an active herpes lesion on the lip. Though the dentist wore gloves, eye protection, and a mask, he was dressed in a short-sleeved shirt. During the procedure, the patient experienced pain and jerked with enough force to knock the explorer out of the dentist's hands. The explorer caused scratches on the arm and penetrated the glove. About 4 days later, the dentist developed herpes whitlow on the hand and arm. He has about four recurrences per year.

### Case #3—Herpes Whitlow on the Neck of a Dental Hygienist

A dental hygienist treated a patient with an active and open herpes lesion on the lip. Although the hygienist wore adequate personal protective equipment (PPE) that included a long-sleeved lab coat, eye protection, face shield, and gloves, the hygienist subconsciously scratched an itch on the back of her neck using her gloved hand. About 4 days later, she developed herpes whitlow on the neck and experiences about four recurrences per year.

### Case #4—Ophthalmic Herpes Keratitis in a Dental Hygienist

A dental hygienist became infected in both eyes with HSV-1. The exact origin of the infection is unknown. That it is work-related cannot be ruled out and is supported by two observations: one, the practice she works for routinely provides hygiene care for patients with active herpes lesions. Two, she regularly performs ultrasonic scaling. With a patient who has active lesions, this is likely to produce a herpes-laden aerosol. The case came to light when others on the dental staff expressed concerns about the potential for cross-infection, since the hygienist was frequently rubbing her eyes while working.

## DISCUSSION

There are over 100 million occurrences of herpes labialis within the United States annually.^5^ Herpes labialis is typically caused by HSV-1 and involves lesions to the lips that, when untreated, resolve in 10 to 14 days.^2^,^3^ Although herpes is highly contagious, the virus is also very sensitive to the use of soap and warm water. Frequent and thorough hand washing will help mitigate risk if the virus is present on the intact skin of the hands.

As evidenced by the four cases cited above, treatment of dental patients who present with active lesions represents a potential occupational hazard to the dental team in the form of herpes whitlow and herpes keratitis.^6^,^7^

Treatment is also problematic for the patient because it risks spread of the lesion and causes discomfort.^1^,^8^ Viral counts are greatest—and thus risk to the dental team is greatest—during open lesion, particularly if there is weeping.^9^ Both a United States Occupational Safety & Health Administration (USOSHA) publication^10^ and published literature^6^ recommend employees with HSV-1 should be restricted from working until the lesions heal. Beyond the use of PPE, no clear-cut standard is offered regarding whether or not to treat a patient with active lesions. Rather, individual dentists make these decisions after giving consideration as to whether elective or urgent treatment is needed.^1^ Some have concluded it is best not to work on patients with active lesions.^6^,^8^

The transmission vector of herpetic whitlow can be via a small cut or abrasion of the skin or finger, or it may be the result of simply touching an active herpetic lesion with the finger.^6^ The infection has serious implications in dentistry. Members of the dental team who are infected create a higher risk for cross-infection to other staff and patients. From a more personal perspective, the pain caused by the infection can be disabling to the point of not being able to work well. Herpes whitlow has been found to occur more frequently in dentists than in the general population (2.4% versus 1.7%; *p* < 0.01).^6^,^11^

Herpes keratitis is typically the result of a person with herpes labialis touching or rubbing the eyes while an active herpes lesion is present on the lips. Another transmission vector exists when a member of the dental team touches his/her eyes after working on a patient with an active herpes lesion. While the literature does not contain information on human viral shedding from the eye during an active infection, there is one study using a validated rabbit model. In untreated eyes, viral shedding took place at 1.4 × 10^7^ plaque forming units (PFU). With acyclovir ointment (3%), shedding was reduced but continued at a substantial rate, 2.3 × 10^2^ PFU.^12^

One potentially under-recognized transmission vector involves an aerosolized saliva/herpes mix. The use of simple safety glasses, even with side shields, may protect against direct spray, but will be of questionable use against an aerosol containing the virus.^13^ HSV-1 has also been found in the saliva of asymptomatic individuals,^8^,^14^ and poses a clear risk for cross-infection.^14^ Harrel and Molinari^15^ concluded that it is reasonable to believe that components of saliva and respiratory fluids are included in aerosols, and that the greatest creators of aerosols in the dental office are the ultrasonic scaler, the handpiece, and the air polisher, respectively.^6^,^15^

As the case histories above illustrate, the use of proper PPE commonly in use for routine procedures is no guarantee of remaining safe. This is particularly true if one fails to wear or circumvents the PPE and creates inadvertent virus-to-skin contact.

## RISK-MANAGEMENT STRATEGIES

Primarily guidelines for infection control procedures originate from the Centers for Disease Control and Prevention^16^ and have been relied on by USOSHA^17^ and the American Dental Association^18^ in development of their own guidelines. These infection control guidelines are based on the concept of universal precautions and focus on blood-borne diseases. They contain little information that is specific to HSV-1. HSV-1 as an occupational hazard for the dental team is not really addressed in depth in these three documents.

Harrel and Molinari^15^ suggest that practitioners should also assume that all patients have infectious disease potentially spread by aerosol, and that this concept should be included as part of the profession's understanding of universal precautions. Further, while the use of PPE eliminates much of the danger from splatter or larger particles, aerosols still have the potential to be inhaled via leaks in the mask and to go around safety glasses. They suggest taking a layered approach to protection. In terms of aerosols, examples include such items as use of high volume evacuators, which capture 95% of aerosols; safety glasses with side shields and a face shield; and goggles.

The authors suggest two general strategies are useful: first, limit treatment of patients with active lesions to urgent care only. Second, in order to minimize disruption of needed dental services, treat active lesions to reduce the length of time it takes them to heal and during which they are infectious. Some larger institutions recommend that routine treatment be delayed for patients with active lesions. For the great majority of dental practices, the dentist will have the ultimate responsibility for setting policies that protect the safety of patients and dental team alike. The following are evidence-based risk-mitigation and management strategies. These are based on the information presented above and even more on the efforts of two expert panels of faculty, one at Dalhouse University and the other at the University of Kentucky. These Practice Guidelines^19^,^20^ were developed following an extensive review of the pertinent literature and made public. They provide the practitioner with very strong evidence. Another strong source is a review of standard infection control procedures with an emphasis on assuring proper fit and use.^13^

Following are risk management strategies for four distinct clinical situations:

**Patients with an active oral herpes infection:**

1. Limit treatment to urgent or emergency care
2. Delay elective procedures until lesions are healed
3. Provide treatment designed to reduce the time of healing
4. Where the use of equipment that produces an aerosol cannot be avoided, use extreme caution and extra PPE that fully covers the body (lab coat or apron), eyes (goggles), and face (facial shield). Disinfect after use
5. Use a National Institute for Occupational Safety and Health N95 rated or higher mask if aerosol may be present (to protect against aerosol contacting oral mucosa)
6. Even if aerosol danger is minimal, wear appropriate extra PPE to cover arms, hands, and to protect face
7. Be aware of the potential for sudden patient reaction to pain. Unless needed, keep the hand that is not holding an instrument out of the “line of fire”
8. Change gloves frequently if the procedure is lengthy, taking care to wash the hands using soap and warm water between glove changes
9. Provide eye protection for the patient and recommend that the patient wash hands and face after treatment
10. Educate the patient on the nature of herpes labialis

**Dental team member has peri-oral herpes infection:**

1. Consider limiting treatment to those who are immune competent
2. Provide treatment designed to reduce the time of healing
3. Cover the lesion area at all times with a suitable mask. An additional covering such as a facial shield will provide additional patient protection
4. Be aware that gloves are now to protect the dental team member from the patient and also the patient from the dental team member. Change gloves immediately if the hand is brought anywhere near the team member's mouth
5. Consider informing the patient of the team member's condition and get their consent to treat

**Dental team member has herpes whitlow:**

1. Whenever possible, because of the highly infectious nature of herpes whitlow, the dental team member should not work until the lesion(s) is healed
2. Provide treatment designed to reduce the time of healing

**Dental team member has herpes keratitis:**

1. Viral shedding potential is high, so face and eyes must be covered to protect the patient. The use of both a face shield and goggles is recommended, as it requires conscious thought and effort to remove them to scratch any itchy eye
2. Change gloves after inadvertent touching of the eyes
3. Wash hands thoroughly with soap and warm water between glove changes
4. Consider informing the patient of the team member's condition and get their consent to treat

For human immune virus (HIV) and hepatitis B virus (HBV), the use of PPE is the accepted standard, and there is no requirement for clinicians to disclose their status. Accordingly, the recommendations offered for consideration above may seem to be overly conservative. One reason is the concern over aerosols, which is fairly unique to dentistry. Another is the difference between blood-borne disease and herpes. It is important to recognize several important differences between HIV, HBV, and HSV-1: first, HIV and HBV are blood-borne diseases. In the dental setting, infection from clinician to patient would occur following direct contact with blood, and is highly unlikely. By contrast, when the clinician has an active HSV-1 lesion, it can be spread by direct contact. The use of PPE will of course make the risk of infection minimal. But as the cases noted above illustrate, these measures are prone to human failings. The highly contagious, highly transmissible nature of HSV-1 means any failure of cross-contamination efforts will likely result in infection. The difficulty of donning gloves without contaminating them when one has a herpetic whitlow lesion is easy to visualize.

Second, a clinician's HIV or HBV status is not evident to patients. Since PPE is not typically worn during nonclinical portions of an appointment, the presence of a herpetic lesion is likely to become evident. Finally, the clinician's HIV and HBV status are long-term issues, while the presence of an active herpetic lesion is a short-term problem.

In terms of avoiding operator to patient infection, in our opinion, a risk–reward analysis is beneficial. Under risk, the authors include both monetary and nonmonetary issues; e.g., pain. For example, if patients are offered an opportunity to reschedule care because a clinician has an active herpetic lesion, there may be disruption of the office's schedule, which affects productivity. However, the risk involved in this sort of disruption relative to a potential negative effect on the patient–doctor relationship if the patient later develops a “sore” should also be considered. Finally, analysis should include consideration of the patient. Providing dental treatment while the patient has an active lesion raises the risk of discomfort, increasing the size of the lesion and delayed healing.

In terms of avoiding patient to operator infections, OSHA regulations create a duty for the owner/operator to take precautions to protect employees.^17^ Here again, risk–reward is an important consideration. The potential risk resulting from the infection of a dental team member is likely to be larger than temporarily foregoing elective treatment for a patient with an active herpetic lesion.
